# Pseudo‐mutual gazing enhances interbrain synchrony during remote joint attention tasking

**DOI:** 10.1002/brb3.3181

**Published:** 2023-07-26

**Authors:** Chun‐Hsiang Chuang, Hao‐Che Hsu

**Affiliations:** ^1^ Research Center for Education and Mind Sciences, College of Education National Tsing Hua University Hsinchu Taiwan; ^2^ Institute of Information Systems and Applications College of Electrical Engineering and Computer Science National Tsing Hua University Hsinchu Taiwan; ^3^ Department of Computer Science National Yang Ming Chiao Tung University Hsinchu Taiwan; ^4^ Department of Computer Science and Engineering National Taiwan Ocean University Keelung Taiwan

**Keywords:** hyperscanning EEG, interbrain synchrony, mutual gaze, cooperation and competition, joint attention, phase locking value

## Abstract

**Introduction:**

Mutual gaze enables people to share attention and increase engagement during social interactions through intentional and implicit messages. Although previous studies have explored gaze behaviors and neural mechanisms underlying in‐person eye contact, the growing prevalence of remote communication has raised questions about how to establish mutual gaze remotely and how the brains of interacting individuals synchronize.

**Methods:**

To address these questions, we conducted a study using eye trackers to create a pseudo‐mutual gaze channel that mirrors the gazes of each interacting dyad on their respective remote screens. To demonstrate fluctuations in coupling across brains, we incorporated electroencephalographic hyperscanning techniques to simultaneously record the brain activity of interacting dyads engaged in a joint attention task in player‐observer, collaborative, and competitive modes.

**Results:**

Our results indicated that mutual gaze could improve the efficiency of joint attention activities among remote partners. Moreover, by employing the phase locking value, we could estimate interbrain synchrony (IBS) and observe low‐frequency couplings in the frontal and temporal regions that varied based on the interaction mode. While dyadic gender composition significantly affected gaze patterns, it did not impact the IBS.

**Conclusion:**

These results provide insight into the neurological mechanisms underlying remote interaction through the pseudo‐mutual gaze channel and have significant implications for developing effective online communication environments.

## Graphical abstract

Pseudo‐mutual gaze channel enables the focus of interacting partners to be shared. EEG hyperscanning and eye trackers are used during remote joint attention tasking. By employing the phase locking value (PLV), we could estimate interbrain synchrony (IBS) and observe low‐frequency couplings in the frontal and temporal regions that differed based on the mode of interaction.

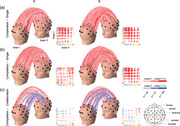

## 1 INTRODUCTION

Social interaction requires eye contact (Jarick & Bencic, 2019; Luft et al., 2022) between individuals. People establish eye contact, perceive nonverbal messages, and express emotions nonverbally to effectively share attention and facilitate mutual understanding. Because of the lockdowns during the COVID‐19 pandemic and recent advances in metaverse technology, people have been embracing remote communication, allowing them to work or study remotely. This modern communication strategy has become indispensable in daily life but is associated with many obstacles due to the lack of eye contact. Therefore, creating a mutual gaze for remote individuals and evidencing the conjunction of eye contact and interpersonal interaction, such as teamwork, decision‐making, and distance educational activity, is of great interest.

Numerous techniques have been proposed to enhance eye contact during videoconferencing. One such method is to simulate mutual gaze, which can be achieved through adjustments such as reorientating the camera at eye level (Microsoft, 2021), minimizing the positional offset between cameras and eyes (Regenbrecht & Langlotz, 2015), and mirroring images to make the remote parties appear to be looking directly at the camera. Studies have used eye trackers to visualize gaze points and have incorporated arrows or gaze‐aware 3D profile photos to indicate who is looking at whom (He et al., 2021; He et al., 2021). By contrast, during a webinar or virtual conference, people typically focus on the shared content displayed on the screen rather than on the camera, and they often listen to the speaker without making eye contact. In these scenarios, individuals may need to be guided on where to direct their attention. If the focus of interacting partners can be visualized and shared, it could lead to a significant improvement in team productivity and efficiency (Dravida et al., 2020; Wohltjen & Wheatley, 2021; Xu et al., 2018).

Many studies have attempted to elucidate single‐person cognitive processes and behaviors. In recent decades, numerous studies have elaborated on the neural mechanisms underlying interpersonal communication activities (Dumas et al., 2010), which represent the theoretical neuroscientific foundation of social interactions. Social neuroscience grounded on hyperscanning techniques (Babiloni & Astolfi, 2014; Czeszumski et al., 2020)—neuroimaging technology for simultaneously exploring the brain dynamics of multiple people—provides deep insights into various social interactions. Imaging modalities, such as electroencephalograph (EEG) (Dikker et al., 2017, 2021; Kinreich et al., 2017), functional magnetic resonance imaging (fMRI), functional near‐infrared spectroscopy (fNIRS) (Dravida et al., 2020), and magnetoencephalography (Dumas et al., 2010; Xu et al., 2018), have been widely used for hyperscanning purposes. Interbrain synchrony (IBS) (Dikker et al., 2017, 2021; Kinreich et al., 2017) between interacting partners is characterized by various connectivity or synchronization analyses (Babiloni & Astolfi, 2014; Czeszumski et al., 2020; Kinreich et al., 2017), including imagery coherency (Nolte et al., 2004), correlation, phase synchrony (Bowyer, 2016; Mormann et al., 2000), and causality (Lachaux et al., 1999; Sciaraffa et al., 2021). Accumulated evidence of increased IBS during hand‐holding (Goldstein et al., 2018), musical performance (Acquadro et al., 2016; Sanger et al., 2012), group activity in classroom (Dikker et al., 2017), movie watching (Kauppi et al., 2010), cooperative and competitive interaction (Astolfi et al., 2010; Babiloni et al., 2007; Dommer et al., 2012; Fallani et al., 2010; Hsu et al., 2021; Sinha et al., 2016), gaming, or decision‐making (Ciaramidaro et al., 2018; Fallani et al., 2010) indicates the neural mechanisms of shared movement and visual coordination.

Joint analysis of eye movement and brain activity has been widely performed in many hyperscanning studies, aiming to uncover neural mechanisms underlying the extent of eye contact in interacting dyads. An fMRI study revealed that eye contact is mediated by the cerebellum and limbic mirror system (Koike et al., 2019). fNIRS signals revealed that eye contact enhances cross‐brain coherence in the angular gyrus (Noah et al., 2020). The higher the level of eye contact of the interacting dyads, the stronger the interbrain coherence in their hemodynamic activities (Dravida et al., 2020). An EEG study elucidated interbrain phase synchronization in the gamma band during eye contact (Luft et al., 2022). However, most of these studies have investigated interbrain coupling during real, in‐person, and direct eye contact. Remote individuals sitting in front of screens and performing joint attention tasks may face challenges in making direct eye contact (George et al., 2022) and in awareness regarding their interacting partner's gaze. Furthermore, a recent study on mother–child interaction (Schwartz et al., 2022) found that connectivity within the temporal regions of two brains was associated with gaze synchrony during direct, face‐to‐face interactions. Such a finding was not observed in the scenario of remote communication. Interestingly, despite weaker IBS, their study suggested brain synchronization could still occur through screen‐mediated interactions. Based on these observations, the current study hypothesizes that incorporating a purposefully designed shared gaze mechanism for interacting individuals could enhance interbrain synchrony and foster the transmission of social signals crucial for accomplishing a joint goal.

In this study, a platform was built that integrated a hyperscanning EEG system and eye trackers, enabling the synchronization of EEG signals and gaze points from interacting dyads. In total, 60 participants were recruited. While performing computer‐based gamelike shared attention tasks, both participants’ eye gaze points would be displayed on their screens (i.e., shared gaze) (Brennan et al., 2008). The participants undertook a target‐searching task in the player‐observer mode and two dual‐player modes: cooperation and competition. The dyads’ gaze behaviors were analyzed, and their IBS, in different modes estimated using a phase locking value (PLV) (Aydore et al., 2013; Lachaux et al., 1999), was compared. This study elucidated that the conjunction of pseudo‐eye contact between remotely interacting individuals can lead to fluctuations in IBS.

Three hypotheses were formulated in this study. First, we proposed that pseudo‐eye contact, facilitated by visualizing the partner's gaze points, could be a nonverbal channel to enhance communication efficiency and enable interacting dyads to adjust gazing patterns to meet both the task's demands and the partner's cues. Second, we hypothesized that the synchrony and desynchrony present in pseudo‐mutual gaze would elicit alterations in the dyad's brain activities to accommodate shifts in social interaction modes. These changes were anticipated to manifest in the IBS of the dyadic frontal, temporal, parietal, and occipital areas, which have been previously reported to be involved in biological motion perception (Saygin, 2007), cooperation and competition (Wang et al., 2022), interoception (Balconi & Angioletti, 2023), informative gesture (Balconi & Fronda, 2020), and have been studied extensively in numerous hyperscanning studies (Czeszumski et al., 2020, 2022; Dravida et al., 2020; Hirsch et al., 2017; Wang et al., 2018; Wikström et al., 2022; Yoshioka et al., 2021). With respect to frequency bands, we hypothesized that elevated IBS might be observed in the delta and theta bands, potentially as results of interpersonal sensory and motor synchrony (Balconi & Angioletti, 2023; Lindenberger et al., 2009) and cooperative joint‐action tasks (Wang et al., 2020). Third, although the current understanding of the influence of dyadic gender composition on IBS is limited, we hypothesized that mixed‐sex and same‐sex dyads could lead to variations in both behavioral performance and IBS. These variations could be particularly substantial in relation to couplings within the frontal–temporal social network, a key area for social interactions (Baker et al., 2016; Li et al., 2021).

## 2 MATERIALS AND METHODS

### 2.1 Ethics statement

This study was approved by the Research Ethics Review Committee of National Tsing Hua University, Taiwan (Registration Number: 11001HT006). All participants signed informed consent forms before the study commencement.

### 2.2 Participants

Sixty undergraduate and graduate students (13 men and 47 women) aged 22.9 ± 2.6 years participated in a two‐person hyperscanning study. The participants were all healthy and had a normal or corrected‐to‐normal vision and normal hearing. None of them were pregnant or chronic smokers, and none had drug or alcohol abuse, physical impairment, psychiatric disorder, or cardiovascular disorder. However, we had to exclude the data from one dyad (consisting of 1 female and 1 male) due to equipment malfunction. This left us with 18 female–female dyads, 10 female–male dyads, and one male–male dyad (Table S1).

### 2.3 Experimental settings

**FIGURE 1:**
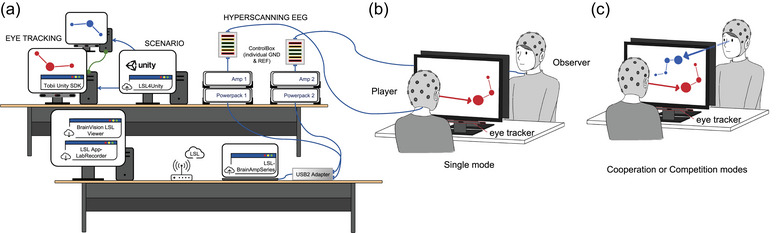
Experimental setup. (a) Hyperscanning setup and multimodal data streaming setting. Each dyad performed the tasks in (b) single‐player mode and (c) cooperation and competition modes.

Figure 1 illustrates the two‐person EEG hyperscanning setting used in this study. Two participants were arranged to sit on both sides of the table. Each side comprised two identical sets of hardware and software, as detailed below.

### 2.4 Hyperscanning EEG recording

This study implemented a 2 × 32‐channel EEG hyperscanning recording mainly by using BrainAmp DC amplifiers and actiCAP slim active electrodes (Brain Products). Two separate electrode boxes, that is, actiCAP ControlBox, between the amplifier and electrodes facilitated individual EEG recording with independent ground and reference. The USB2 Adapter served as an EEG acquisition hub, synchronizing amplifiers and linking to the recording computer through fiber optic cables. The 32 electrodes on the EEG cap (Easycap) were arranged according to the extended International 10–20 system to record signals at the sites of Fp1, Fz, F3, F7, FT9, FC5, FC1, C3, T7, TP9, CP5, CP1, Pz, P3, P7, O1, Oz, O2, P4, P8, TP10, CP6, CP2, Cz, C4, T8, FT10, FC6, FC2, F4, F8, and FP2. The ground and reference electrodes were positioned at the scalp sites of Fpz and FCz, respectively. Conductive gel (SuperVisc Easycap) was applied between the electrodes and scalp to maintain impedance below 10 kΩ. The sampling rate was 1000 Hz, and the DC resolution was 24 bits.

### 2.5 Pseudo‐eye contact

During hyperscanning recording, each dyad was restricted from having any form of communication while performing joint attention tasks. The two participants sat across from each other, facing their own computer screens (ViewSonic VA2756‐MH 27″), with no visibility of each other's faces or gestures, and could not talk. The computer screen was the only channel through with they could interact. To enable such communication, this study proposed a virtual eye‐to‐eye communication channel by using two eye‐tracking devices (Tobii Eye Tracker 5; Tobii Technology AB). The gaze point of each participant was tracked separately and continuously. The gaze points mirrored onto computer screens were rendered as letters “A” and “B” to differentiate between participants and enable pseudo‐eye contact. The *x*–*y* coordinates and the corresponding timestamps of the gaze points were recorded.

### 2.6 Data streaming and synchronization

In this study, a data streaming platform was built on the basis of LabStreamingLayer (LSL) (Swartz Center for Computational Neuroscience, UCSD) (Kothe et al., 2019) to synchronize time‐series recordings, experiment events, and participants’ behaviors recorded from different devices. The EEG hyperscanning data captured from two participants of a dyad were streamed using the BrainAmp Series LSL connector and monitored using the BrainVision LSL Viewer (Kreilinger, 2020). The main user interface was built using the Unity game engine (Unity Technologies). This Unity‐based Interaction Unit served as a stimulus presentation hub, rendering 2D gaming scenarios for joint attention tasks (detailed in the next section), handling I/O and event markers, and interacting with the Tobii Unity SDK for eye‐tracking. The custom streaming app served as a bridge of the mutual communication between Unity and LSL. All data streams on the same local network were synchronized over the LSL at submillisecond accuracy. The data recorder, namely, App‐LabRecorder (Kothe et al., 2019), centralized all data streams with a unified time domain in a single eXtensible Data Format (.xdf) file.

### 2.7 Shared attention tasks

**FIGURE 2:**
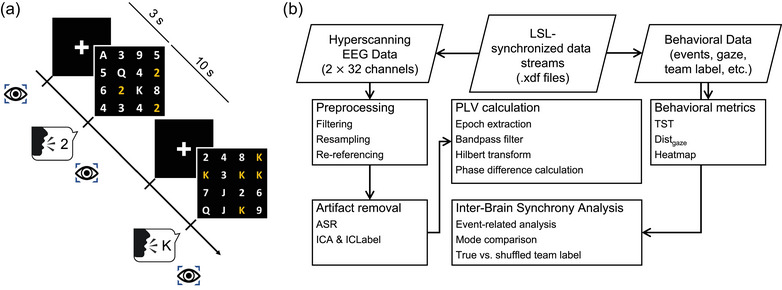
(a) Experimental paradigm. (b) Data analysis flowchart.

This study investigated the dynamics of joint attention between two participants through a visual search game that required gazing. The game presented both participants with an identical sequence of visually and acoustically presented words (Figure 2a). The screen displayed a 4 × 4 matrix containing 16 alphanumeric characters, with one, three, or five targets matching the acoustically presented words. The auditory stimuli consisted of synthetic human voices saying “Ace, Two, Three, …, Jack, Queen, and King,” whereas the matrix displayed on the screen was composed of 16 alphanumeric characters selected from [*A*, 2, 3, …, 10, *J*, *Q*, or *K*]. Each trial began with a 3‐s fixation cross, followed by 300 ms of the synthetic human voice. Participants then had 10 s to search for all the target(s) in the 4 × 4 matrix of alphanumeric characters.

The eye tracker was the only device used for interaction in the game. Before the experiment, the participants followed Tobii's calibration procedure instructions. During the task, the participants tried to locate the target by maintaining their gaze on it for more than 700 ms, which caused the marked target to turn red. The participants then continued to search for any other possible targets. Once all targets were marked, the participants were instructed to gaze at the fixation point located at the bottom right corner of the screen. The gaze points of both participants were displayed as two differently colored circles on the screen.

### 2.8 Single‐player, competition, and cooperation modes

The game was played in three modes: player‐observer, two‐player competition, and two‐player cooperation modes. In the single‐player mode, two participants played in rotation as the player and observer, and only the player's gaze was displayed on both screens. In the competition mode, two participants raced to be the first to identify targets. Each target could only be marked once, and the more targets marked, the higher the score. In the cooperation mode, the two participants marked the targets together by gazing at the same target simultaneously for >700 ms to score a point. In both dual‐player modes, two screens displayed both participants’ gazes, making them aware of each other's gaze points during the task. Additionally, each trial (also known as “round”) lasted for 13 s, each mode included 20 trials, and each dyad played the game in each mode twice in a counterbalanced order.

### 2.9 Behavioral data analysis

The participants’ time required for searching targets and the distance between two participants’ gaze points in the three modes were calculated as follows.

The first target‐searching time (TST) was the time elapsed between the stimulus onset and first target‐locked onset (defined as 700 ms before target identification). The remaining TSTs were the time elapsed between two consecutive target‐locked onsets. The means of TSTs were calculated by searching the first to the fifth target while trimming 10% outliers for further analyses.

Assuming that the two participants’ interactions while performing the task could be understood through virtual visual contact, this study quantified the extent to which their virtual visual contact by calculating the distance between gaze points (abbreviated as Dist_gaze_). Namely, the Euclidean distances between the *x*–*y* coordinates of two participants’ gaze points were calculated. Additionally, the dyad's gaze movements in searching targets were visualized using heatmap. The similarity between gaze heatmaps could be estimated using Pearson's *r* coefficient, structural similarity index measure (SSIM), and Jaccard similarity coefficient.

### 2.10 EEG data preprocessing

Figure 2b shows the data analysis flowchart. The EEG hyperscanning data recorded from two participants were separated, downsampled to 250 Hz and filtered with a 1–50‐Hz band‐pass finite impulse response filter, and followed by Artifact Subspace Reconstruction (cutoff parameter *k* = 20) (Chang et al., 2019; Hsu et al., 2021) to remove high‐amplitude artifacts. Considering the gaze‐centric focus of this study, there was a high expectation of eye artifacts impacting the frontal electrodes. To prevent any potential spread of noise to less‐affected electrodes, we opted to re‐reference the EEG signals to mean mastoids, that is, the average of TP9 and TP10. Being distanced from the primary sources of eye movement artifacts, these sites offered a more appropriate choice for the current study. Furthermore, to manage artifact rejection, we utilized independent component analysis (ICA, infomax algorithm) (Makeig et al., 1995) and ICLabel (Pion‐Tonachini et al., 2019). Each EEG component, decomposed by ICA, underwent assessment via ICLabel to establish its probability of being associated with brain, muscle, eye, heart, line noise, channel noise, or other sources. Leveraging these computed probabilities, we discarded non‐brain components with a likelihood exceeding 90% of being categorized as muscle, eye, heart, line noise, channel noise, or other sources. This 90% threshold aligns with that utilized in Delorme's “Optimized Pipelines” (Delorme, 2023) and other studies (Bierwirth et al., 2023). The remaining independent components were then back‐projected to complete the signal reconstruction. The whole artifact removal process was executed automatically using native and plug‐in functions of the EEGLAB toolbox (v2020.0) (Delorme & Makeig, 2004) on MATLAB (R2020a). Note that both Artifact Subspace Reconstruction and ICA are component‐based methods that remove artifact components via a linear transformation without excluding any segments from the EEG epoch. As a result, the average signal length for each condition was preserved, remaining identical to the original data length. Before the estimation of interbrain connectivity, the preprocessed data were segmented into uniformly timed epochs. These epochs were time‐locked to the voice stimulus, spanning from 3 s prior to, up to 10 s following the stimulus.

### 2.11 Interbrain connectivity

This study employed PLV (Lachaux et al., 1999), a broadly used frequency‐specific synchronization measure of EEG signals, to assess interbrain connectivity. Specifically, all EEG signals were processed with a band‐pass filter to extract signals with desired frequency components, where the passbands included *δ* (1–3 Hz), *θ* (4–7 Hz), *α* (8–12 Hz), *β* (13–30 Hz), and *γ* (31–50 Hz) bands. Next, the Hilbert transform was used to estimate the instantaneous phases of all filtered signals, resulting in time courses of signal phase angles ranging between –*π* and *π*. The PLV between pairwise channels was obtained by averaging the absolute value of the phase differences between signals over the trials. The original PLV ranged from 0, implying an absent phase synchronization, to 1, implying a perfect phase synchronization. Note that although this study did conduct a control analysis (see Section 2.12) for spurious interbrain couplings, one should be aware of volume conduction effects (Aydore et al., 2013; Bruña et al., 2018) on the interpretation of the obtained IBS.

Numerous previous hyperscanning studies (Czeszumski et al., 2020, 2022; Dravida et al., 2020; Hirsch et al., 2017; Wang et al., 2018; Wikström et al., 2022; Yoshioka et al., 2021) have underscored that most brain regions are implicated in social behaviors to varying degrees. Therefore, this study calculated PLV for all combinations of channel pairs. To facilitate interpretation of the results, the 32 EEG electrodes were grouped into five major regions: frontal (Fp1, Fp2, F7, F3, Fz, F4, F8, FC5, FC1, FC2, and FC6), central (C3, Cz, and C5), temporal (FT9, T7, TP9, FT10, T8, and TP10), parietal (CP5, CP1, CP6, CP2, P7, P3, Pz, P4, and P8), and occipital (O1, Oz, and O2).

### 2.12 Statistical analysis

This study employed one‐way repeated‐measures ANOVA, with Holm–Bonferroni post hoc tests, to determine whether there were significant differences in TST, gaze behavior, and PLV between the three task modes. To eliminate the concern that IBS was elicited by the experimental environment rather than by eye contact, virtual eye contact‐associated interbrain connectivity was examined in each channel by comparing the PLV between the participants of the true team label (PLV_true_) with that of the shuffled team label (PLV_shuffled_). Given that this study included 29 dyads, 29 PLV_true_ values, and 1624 (58×56/2) PLV_shuffled_ values per channel pair were obtained. This study used the confidence interval approach with the bootstrap method for each pair to evaluate whether the PLV_true_ value significantly differed from the PLV_shuffled_ value. The bootstrap distributions of the mean PLV_shuffled_ values were built, where each distribution contained 1000 bootstrap sample means, with each mean calculated from 200 random samples. All the multiple comparisons were corrected using the false discovery rate. This study further incorporated the factor of dyadic gender composition to examine it is effect on TST, gazing pattern, and IBS. The statistical model was the mixed factorial ANOVA with task mode as the within‐subjects factor and gender composition as the between‐subjects factor. The task mode consisted of three levels: single, cooperation, and competition. The gender composition involved two categories: female–male and female–female dyads. The lone male–male dyad was not included in the analysis.

## 3 RESULTS

### 3.1 Behavioral performance

**FIGURE 3:**
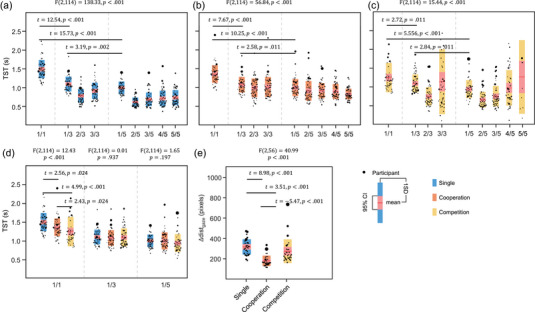
Behavioral analysis. The participants’ target‐searching time (TST) in (a) single, (b) cooperation, and (c) competition modes. (d) Comparison of the searching time for the first target among task modes in one‐, three‐, and five‐target trials. (e) Distance between participants’ gaze points in the different task modes.

Figure 3 illustrates the distributions of TSTs recorded in the single, cooperation, and competition modes. Each dot represents a single participant's mean TST. The dot radius varied with the absolute value of the *z*‐score normalized TST, representing behavioral variations. As an overarching trend, the number of targets significantly influenced the TST of the first target across all task modes: single (Figure 3a, *F*(2, 114) = 138.33, *p* < .001), cooperation (Figure 3b, *F*(2, 114) = 56.84, *p* < .001), and competition (Figure 3c, *F*(2, 114) = 15.44, *p* < .001). In all three task modes, the one‐target condition exhibited a significantly prolonged TST for locating the first target in comparison to the three‐ and five‐target conditions (all *p*s ≤ .011).

In single‐player mode under the three‐ or five‐target conditions, the time taken to locate the first target (denoted as 1/3 and 1/5 in Figure 3a) was significantly longer than the time taken to locate subsequent targets (denoted as *x*/3 and *x*/5, where *x* ≠ 1). For the three‐target condition, this difference was statistically significant (*F*(2, 110) = 54.57, *p* < .001), with post hoc comparisons indicating that the time taken to locate the first target was longer than for the third target, which in turn was longer than for the second target (1/3 > 3/3 > 2/3, *p*s < .001). For the five‐target condition, the difference was also significant (*F*(4, 220) = 52.62, *p* < .001), with post hoc comparisons revealing that the time taken to locate the first target was longer than for any subsequent target (1/5 > *x*/5, *p*s < .001), and the time taken to locate the second target was shorter than for the fourth and fifth targets (2/5 < 4/5, 5/5, *p*s ≤ .002).

In contrast, in the cooperation mode (Figure 3b) under the three‐target condition, there was no significant difference in the TST for searching different targets (*F*(2, 110) = 2.21, *p* > .05), indicating that the time spent did not depend on the order of the targets being searched for. In the case of the five‐target condition under the cooperative mode, the only significant difference was observed between the first and the last targets (*F*(4, 220) = 5.36, *p* < .001; post hoc: 1/5 > 5/5, *p* < .001).

In the competition mode (Figure 3c), the TST exhibited a U‐shaped curve in the three‐ and five‐target conditions. Specifically, for the three‐target condition, the first and third targets took significantly more time than the second target (*F*(2, 82) = 7.15, *p* < .001; post hoc: 1/3, 3/3 > 2/3, *p*s < .01). In the five‐target condition, the fifth target had significantly longer TST compared to the second and third targets (*F*(4, 76) = 5.49, *p* < .001; post hoc: 5/5 > 2/5, 3/5, *p*s ≤ .003). These results suggested that the participants spent the shortest TST searching for the second target. The dots became sparse, particularly when searching for the fifth target because the likelihood that a single participant identified all five targets was low.

Figure 3d provides a comparison of the TSTs of the first target between the three modes. In the one‐target condition, the three modes exhibited significantly different TSTs (*F*(2, 114) = 12.43, *p* < .001). The shortest TST was observed in the competition mode (1.236 ± 0.395 s), followed by the cooperation mode (1.368 ± 0.236 s) and the single‐player mode (1.507 ± 0.245 s) (post hoc: *p*s ≤ .024). However, no significant differences in the time to identify the first target were noted in the three‐ (*F*(2, 114) = .01, *p* = .937) and five‐target (*F*(2, 114) = 1.65, *p* = .197) conditions.

**FIGURE 4:**
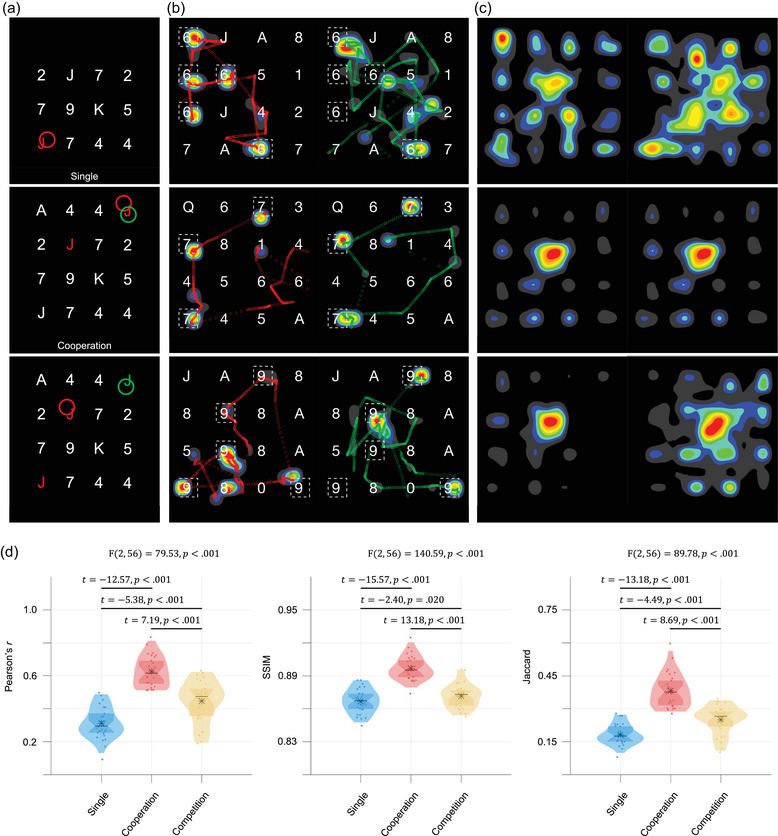
Gaze movements. (a) Examples of participants’ eye gazes in three modes. There are 16 alphanumeric characters, represented by a 4 × 4 matrix, displayed on the screen. Each trial had one, three, or five targets out of 16 characters matching the acoustically presented words. In the single‐player mode, the red circle displayed on the screen indicates the participant's gaze point. In the cooperation and competition modes, the two participants’ gaze points on the screen are distinguished by red and green circles. (b) Examples of participant's scan paths and gaze heatmap observed in a single trial. The dashed box, which was invisible during the experiment, labels the positions of the target. Note that the observer's gaze point was invisible during the experiment. (c) Gaze heatmaps of 40 trials. (d) Similarity between the two participants’ eye‐tracking heatmaps. Each violin plot (transparent color) overlaid with a box plot displays the distribution of the similarity measures, including Pearson's *r*, structural similarity index measure (SSIM), and the Jaccard similarity coefficient, in single, cooperation, and competition modes. The black asterisk and line indicate the mean and median of the similarity, respectively. The significant difference in the similarity between task modes was determined using repeated‐measure ANOVA.

During the task, the eye gaze point(s) of the participant(s) were displayed on the screen(s) in real‐time (Figure 4a). In the single‐player mode, only the player's gaze point was visible to the dyad. Figure 4b illustrates the dyad's scan paths and gaze heatmaps for a single trial, whereas Figure 4c shows the gaze heatmap for 40 trials. In the two‐player mode, the gaze points of both participants tended to wander around the screen center compared to the single‐player mode.

Figure 4d presents three analyses reflecting the similarity between the gaze heatmaps of the dyads. Take the Jaccard similarity coefficient as an example, a significant difference was observed across the three modes in the dyad's gaze patterns (*F*(2, 56) = 89.78, *p* < .001). The dyad's gaze patterns were largely congruent in the cooperation mode, largely incongruent in the single‐player mode, and intermediate in the competition mode (post hoc: *p*s < .001).

Additionally, the distance between dyadic gaze points, Dist_gaze_ (in pixels), exhibited a significant difference across the three modes (Figure 3e) (*F*(2, 56) = 40.99, *p* < .001). The shortest Dist_gaze_ (178.9 ± 52.7 pixels) was observed in the cooperation mode. In the competition mode, two participants competed for searching for targets, leading the two gaze point trajectories recorded from the participants to be repulsive and the resultant Dist_gaze_ (272.7 ± 118.2 pixels) to be significantly large. The largest Dist_gaze_ (313.4 ± 78.0 pixels) was found in the single‐player mode (post hoc: *p*s < .001).

### 3.2 IBS during pseudo‐eye contact

**FIGURE 5:**
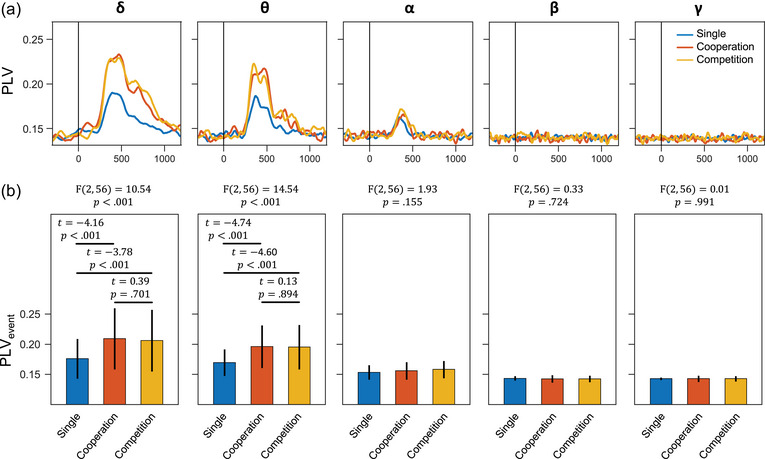
Phase locking values (PLVs). (a) PLV time courses of three modes, where each time course was averaged across all channel pairs. The baseline correction was applied to all time courses. (b) Comparison of event‐related PLV (PLV_event_) among three modes, where PLV_event_ were averaged from 200 to 500 ms.

Figure 5 presents the IBS connectivity changes, estimated using interbrain PLV, of the three modes in the five frequency bands, where each PLV time course was averaged across all channel pairs. The results (Figure 5a) revealed that the *δ*‐, *θ*‐, and *α*‐PLVs of the three modes significantly increased after the onset of the stimulus (*p*s < .001). Furthermore, the task mode had a significant effect on the event‐related PLVs (PLV_event_) (Figure 5b), particularly in the *δ*‐ (*F*(2, 56) = 10.54, *p* < .001) and *θ*‐ (*F*(2, 56) = 14.54, *p* < .001) bands. Post hoc comparisons indicated that PLV_event_ observed in the cooperation modes were significantly higher than those in the single‐player mode (*p*s < .001).

**FIGURE 6:**
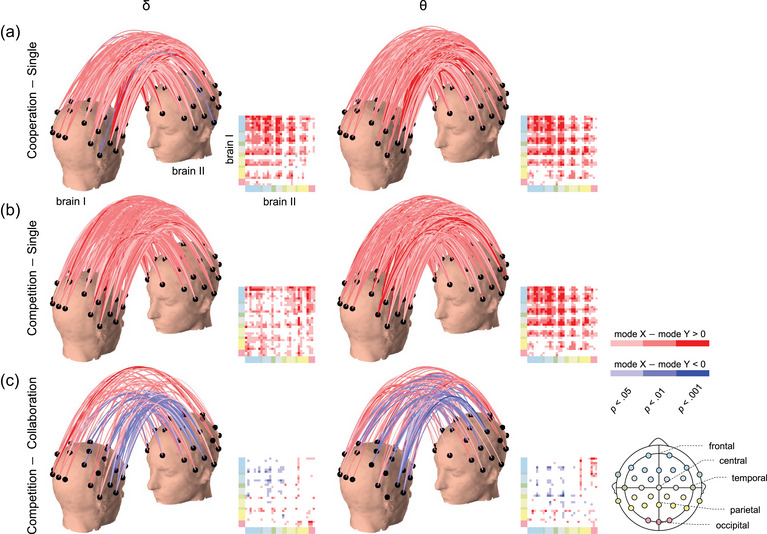
Comparison of each interbrain connection among task modes. Differences in phase locking value (PLV) between (a) cooperation and single‐player modes, (b) competition and single‐player modes, and (c) competition and cooperation modes. The reddish and bluish edges indicate that the differences between the two modes are significantly positive and significantly negative, respectively.

Figure 6 presents all significantly different IBS connections between task modes in the *δ*‐ and *θ*‐bands. For most connections involving the frontal, central, and parietal regions, the PLVs of the cooperation and competition modes were significantly higher than those of the single‐player mode, indicating increased IBS coupling associated with dyads’ eye contact (Figure 6a,b). Some significant differences were noted in interbrain PLVs between the cooperation and competition modes: significantly stronger IBS couplings in the frontal–central regions were found in the cooperation mode, whereas significantly stronger IBS coupling in the temporal and occipital regions was found in the competition mode (Figure 6c).

### 3.3 Interbrain PLV of true and shuffled team labels

**FIGURE 7:**
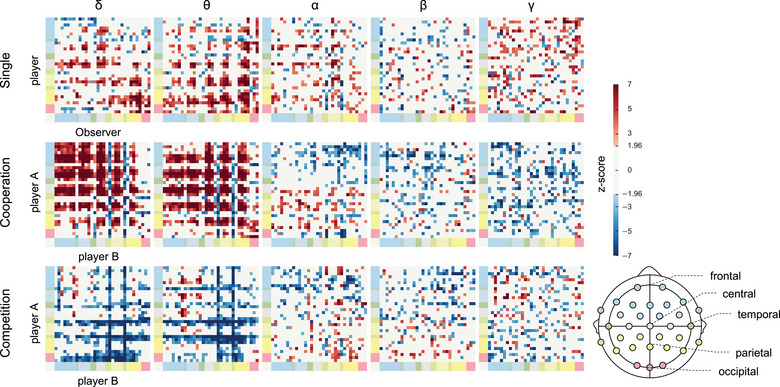
Comparison of interbrain phase locking values (PLVs) between true and shuffled teaming labels. The bluish and reddish color is the *z*‐score, which shows the number of standard deviations that the PLV_true_ lies from the mean of PLV_shuffled_. The white color exhibits a statistically nonsignificant difference. The colors shown on both axes indicate the channel locations.

This study compared the PLVs of the true team label to those of the shuffled teaming label. If the PLVs of the true team label differed significantly from those of the shuffled team label, the coupling between brain regions would be considered a “valid” interaction connection. Interaction‐related IBS could be used as an effective indicator to differentiate the team label. Figure 7 illustrates the significant PLV of the three modes in the five frequency bands. Overall, the EEG connectivity in the *δ* and *θ* bands indicated region‐specific and mode‐specific IBS coupling or decoupling.

In the single‐player mode, several IBS couplings in the *δ*‐band were identified in the frontal–central and parietal–parietal connections. More IBS couplings were identified in the *θ*‐band, particularly for the connections associated with the parietal and central regions. The cooperation mode revealed strong IBS coupled in the *δ* and *θ* bands among most regions of the brains of the dyad, except for the temporal and occipital areas. However, IBS decoupled in the *δ* and *θ* bands in the connection associated with temporal regions. Strong IBS decoupling occurred in the competition mode. Similar to the cooperation mode, most decoupled phenomena were identified in the connection associated with temporal regions. However, IBS coupling was rarely found in the competition mode.

### 3.4 Gender composition effects on TST, gaze trajectory, and IBS

**FIGURE 8:**
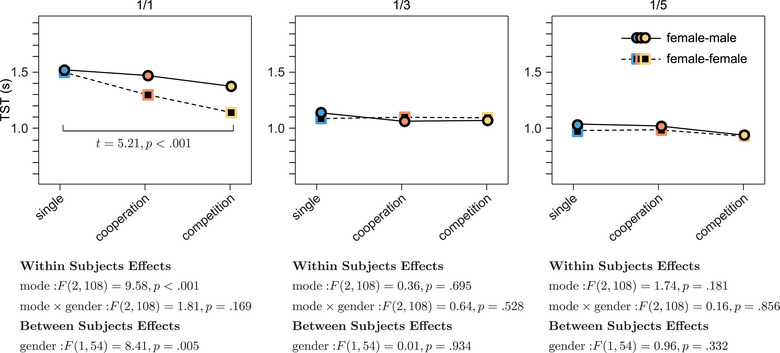
Comparison of target‐searching time (TST) for female–male and female–female dyads across single, cooperation, and competition modes under one‐, three‐, and five‐target conditions. The notations, 1/1, 1/3, and 1/5, represent the first‐target under one‐, three‐, and five‐target conditions, respectively.

First, a mixed factorial ANOVA with task mode as the within‐subjects factor and gender composition as the between‐subjects factor was employed to assess their impacts on TST. As demonstrated in Figure 8, the task mode significantly influenced the TST exclusively in the one‐target condition (mixed factorial ANOVA, *F*(2, 108) = 9.58, *p* < .001), a finding that aligned with the results presented in Figure 3d. Moreover, gender composition significantly impacted the TST in the one‐target condition (*F*(1, 54) = 8.41, *p* = .005), whereas it had no significant effect in the three‐ and five‐target conditions. However, across the different target conditions, there was no significant interaction effect between the task mode and gender composition on the TST.

Second, the similarity of participants’ gaze heatmaps across different task modes was also evaluated via a mixed factorial ANOVA, incorporating the dyadic gender composition as a categorical factor. As displayed in Table 1, the results demonstrated that although task mode significantly influenced gaze patterns, the gender composition did not.

**TABLE 1:** Effects of task mode and gender composition on the similarity of participants’ gaze heatmaps.

Mixed factorial ANOVA	Pearson's *r*	SSIM	Jaccard
Between subjects effects			
gender composition	*F*(1, 26) < .01	*F*(1, 26) = .07	*F*(1, 26) = .14
	*p* = .983	*p* = .793	*p* = .871
Within subjects effects			
task mode	*F*(2, 52) = 67.10	*F*(2, 52) = 122.96	*F*(2, 52) = 26.15
	*p* < .001	*p* < .001	*p* < .001
task mode × gender composition	*F*(2, 52) = .17	*F*(2, 52) = 2.76	*F*(2, 52) = .48
	*p* = .845	*p* = .073	*p* = .751

Abbreviation: SSIM, structural similarity index measure.

**FIGURE 9:**
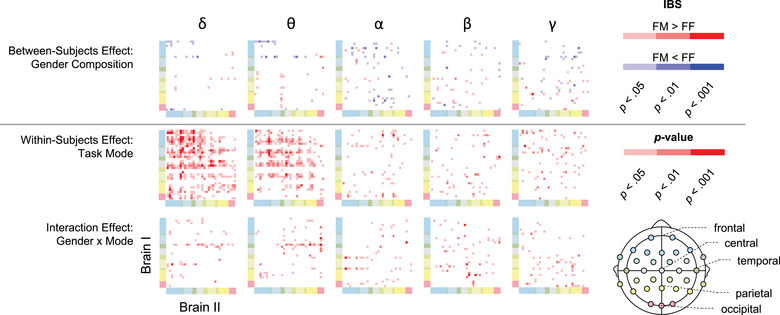
Effects of task mode and gender composition on interbrain synchrony (IBS). The terms FF and FM denote female–female and female–male dyads, respectively.

Third, the effect of dyadic gender composition on IBS is depicted in Figure 9. While the findings suggested that gender composition did have an effect on certain channel pairs, these effects manifested in a scattered pattern, as seen in the first row of the figure. Regarding the within‐subjects effects, task mode showed significant influence on both delta and theta IBS, which aligned with the findings shared in Figure 7. Notably, an interaction effect between gender composition and task mode was observed on IBS but in a limited number of channel pairs.

## 4 DISCUSSION

In summary, the study proposed a novel way for interacting partners to share their attention and focus during remote tasks without verbal communication using a pseudo‐eye contact solution. The study used a hyperscanning EEG system to scan the brain activity and analyzed interbrain couplings of dyads while they performed a visual search task in single‐player, cooperation, and competition modes. The results showed that the dyads’ IBS increased in the frontal and central delta and theta bands when the task involved pseudo‐eye contact. Additionally, the IBS coupling and decoupling were associated with different eye‐gazing patterns, with IBS coupling occurring during cooperation and IBS decoupling occurring during competition.

Compared with multiple targets, the TST for one target was the longest. This finding reasonably reflects the difficulties in finding a single object among a group of distractors. The prevalence effect (Wolfe et al., 2005), referring to declined behavioral performance due to low prevalence, can explain the relatively long TST. As the number of targets increased, the TST for the first target reduced, which is consistent with the results of previous studies (Gorbunova et al., 2019). As expected, the TST rebounds as the number of remaining targets decreases.

In the cooperation mode, a shared gaze enables interacting partners to easily know where their partner is looking and to identify where the target of mutual interest is located. Shared gaze reduces the cost incurred by the spatial referencing time (Neider et al., 2010), leading interacting partners to identify the spatial information of the target efficiently. This nonverbal communication using shared gaze for spatial referencing time can be even more efficient than verbal communication (Brennan et al., 2008). Consistent with these findings, the time spent searching for designated targets was significantly reduced when interacting partners were working together.

However, this phenomenon occurs only in the single‐target condition. In the multitarget conditions (Figure 3b), the differences in TSTs for the first and subsequent targets were nonsignificant. Compared with the single‐player mode, these TSTs were even longer in the cooperation mode. This difference in TST might represent the time cost for interacting dyads to agree on selecting common targets. The participants might cooperate with their partners to reduce conflict (Evans et al., 2015), synchronize their gaze, and identify targets that cost extra time for coordination (D'Angelo & Gergle, 2018).

The TST was significantly the shortest when the participants were competing for only one target. However, as the searching task became less difficult, the three modes exhibited no significant difference in TST for the first target in the multitarget conditions (Figure 3d). Notably, the participants could rapidly identify the second target, but not the subsequent targets (Figure 3c). This might be related to spatial working memory while performing visual search tasks (Attar et al., 2016; Oh & Kim, 2004). The participants might rapidly glance around the letters on the screen and memorize the target locations, enabling them to efficiently identify the second target. This spatial working memory skill meets the needs of current visual search tasks. However, the capacity to retain the spatial locations of targets is limited. Moreover, as the opponent locates more and more targets, the number of available targets is reduced. The task becomes increasingly challenging, resulting in declining behavioral performance that is even worse than that observed in the single‐player mode. These results are consistent with previous findings that competition affects working memory performance (DiMenichi & Tricomi, 2015). Other studies (DiMenichi & Tricomi, 2016) have also concluded that decreased working memory performance during competition can be predicted by increased brain activity.

During social interaction, eye contact is one of the key communication channels that can coordinate shared attention between interacting partners (Wohltjen & Wheatley, 2021), enhance arousal (Jarick & Bencic, 2019), increase engagement (Kompatsiari et al., 2018), and facilitate social presence (Macdonald & Tatler, 2018). This study used commercial eye‐tracking devices with an in‐house live streaming technique to enable interacting dyads to see their interacting partner's gazing point on the screen in real time. In the current experiment, the location of the partner's gaze, visualized as a cursor, was visible on the screen. The participants could interact seamlessly with remote partners through pseudo‐eye contact. Similar to previous studies, involving codriver (Maurer et al., 2014) and visual searching and matching tasks (Jing et al., 2021), the current tasks requiring coordinating spatial referencing and attention benefit from this shared gaze.

Many studies (D'Angelo & Schneider, 2021; Li et al., 2016) have used shared gaze for cooperative and competitive activities such as online gameplay. The heatmaps of the gaze points offered in this study showed that the interacting parties could achieve the required tasks by sharing their visual focus. Three similarity metrics, namely, Pearson's *r*, SSIM, and Jaccard, confirmed that dyads’ eye contact varied with the task mode. During cooperation, the dyads’ eye trajectories highly overlapped, and during competition, their eye trajectories tended to be mutually exclusive. This discrepancy was attributed to the nature of the task instruction. Cooperative interaction required efficient behavioral coregulation between partners, whereas competitive interaction met the goal of defeating the opponent. In the single‐player mode, the observers observed the player searching for targets but randomly glanced at the screen most of the time, leading to the largest dissimilarity of the eye gaze patterns between the dyads. These results confirmed that the embodied simulation (Bergen, 2012) established between interacting parties through the shared gaze contributed to a mutual understanding of intention. The proposed pseudo‐eye contact for shared gaze could enable interactive activities.

The design of gaze visualization was found to affect cooperative performance and coordination time (D'Angelo & Gergle, 2018; Li et al., 2016). The eye movement patterns visualized in either the heatmap, shared area, or path could support the coordination between collaborators. Different forms of gaze visualization may be involved with a variety of IBS. Further neuroscientific evidence is required to obtain an optimal gaze‐based intervention without distracting features.

In the cooperation mode, both participants were required to identify all targets. One needed to rapidly identify targets first, and the other should keep up with the partner's eye movement. The former and the latter are considered the leader and the follower, respectively. A well‐established leader–follower relationship in interacting dyads contributed to behavioral synchrony (Jiang et al., 2015), that is, highly‐overlapped eye movements between the participants. Notably, the participants could perceive the roles they best played. They were neither assigned the role of leader or follower before the experiment nor allowed to discuss it during the experiment. The roles of leader and follower naturally emerged in the presence of shared gaze. In IBS connectivity, interacting dyads presented with enhanced phase synchrony in the *δ* and *θ* bands. In line with previous findings (Bilek et al., 2015; Dravida et al., 2020; Noah et al., 2020), this slow‐frequency interbrain connectivity is related to the neural mechanism of leader–follower relationships in cooperation. Slow‐frequency synchrony has also been reported in other nonverbal communication, such as observation of social gestures (Balconi & Fronda, 2020).

This phenomenon is evident between the speaker and listener (Pérez et al., 2017) during verbal communication. The synchronized behavior, modulated by the top–down process of mutual prediction and adaptation (Konvalinka et al., 2010), leads to phase synchrony in EEG. Corollary discharge theory (Guthrie et al., 1983; Subramanian et al., 2019) supports the contribution of the shared gaze to cooperative behaviors. The interacting dyads’ synchronized eye movements manifested as their visual and motor systems accurately detecting and tracking the fast motion of their partner's saccades. The alteration of frontal cortex neurons by the corollary discharge signal (Ford & Mathalon, 2004; Fronda & Balconi, 2020; Sommer & Wurtz, 2006; Sommer & Wurtz, 2008) might explain the current interbrain couplings underlying the shared gaze predominantly emerging in the dyad's anterior brain areas.

IBS enables the roles of leader and follower to be distinguishable. Connectivity analyses (Balconi & Fronda, 2020; Sänger et al., 2013) have demonstrated that increased interbrain coupling emerged from leaders to followers. Although the frontal *α* (Konvalinka et al., 2014) can be used to distinguish leaders from followers, most of the current *α*‐band IBS were nonsignificant during the cooperation mode, likely due to the leader–follower role interchange throughout the experiment.

Compared with the single‐player mode, the competition mode (Figure 6) elicited an increased slow‐frequency IBS. Similar to cooperative interaction, increased coupling between the brains of the dyad account for motor coordination processes and behavioral coregulation between participants (Barraza et al., 2020). Such coupling denotes varying degrees of increases in competition and cooperation modes. Several studies (Sinha et al., 2016) have demonstrated increased interbrain connectivity during competition, but some (Barraza et al., 2020) have reported opposite results. Accordingly, the difference between the two modes observed in the frontal area presents a stronger IBS while cooperating. Conversely, IBS involving the temporal, parietal, and occipital areas is stronger while competing. Regardless of social interactions, an increased low‐frequency IBS might be an EEG signature for reasoning someone's intentions and reaching goals during competition and cooperation.

Accumulating evidence (Czeszumski et al., 2020; Wang et al., 2018; Yoshioka et al., 2021) indicates the role of the temporoparietal junction in social interaction. In the present study, the significantly decreased *δ*‐ and *θ*‐IBS observed in the temporal area (Figure 7), particularly in the competition mode, indicates the process of self–other distinction (Koster‐Hale et al., 2017). The absent frontal connectivity and reduced temporal connectivity suggest that the participants might adopt some self‐focused strategies to defeat the opponent.

The shared gaze enabled the participants to accurately convey actions and ideas to their partners, receive and understand their intentions, and adapt behavior to achieve seamless interactions. Notably, people who are exposed to the same or even different stimuli can exhibit similar event‐related brain responses in their EEG signals (Valencia & Froese, 2020), leading to fake interbrain coupling. As with the approaches adopted in previous studies (Bilek et al., 2015; Dravida et al., 2020; Koike et al., 2019; Noah et al., 2020), this study examined the existence of IBS by comparing the PLV between real and shuffled dyads and concluded that a shared gaze promoted neural couplings between remote people.

Studies (Dravida et al., 2020; Hirsch et al., 2017; Noah et al., 2020) have indicated that brain connectivity increases with in‐person eye contact. The present couplings across brains were anchored in social interactions and shared gaze (Kinreich et al., 2017). Communication between remote participants benefits from pseudo‐eye contact, which can result in more synchrony in brains. The proposed approach for sharing gaze combined with EEG hyperscanning is ideal for studying the neural mechanisms underlying interaction behavior.

Several limitations of this study should be noted. We aimed to replicate remote interactions using the current experimental setup, yet a key limitation stems from the close physical proximity of participants during the experiment. Despite participants being unable to perceive each other visually, their proximity may have allowed the exchange of subtle cues, such as body temperature, respiration rate, and body odors. These are signals typically absent in authentic remote interactions, and their presence could have influenced the degree of synchrony observed in our EEG data. Although we controlled for the most overt external influences, these subtler factors might have contributed to an environment that, in some respects, more closely resembles a face‐to‐face interaction rather than a remote one. As such, these factors should be considered when interpreting our results, as they might have led to an overestimation of the level of synchrony that would be achieved in a truly remote interaction. In future research, it could be beneficial to physically separate participants, potentially in different rooms, to mimic real remote interactions better and eliminate these subtle nonvisual cues. Doing so would help refine our understanding of neural synchrony in remote interactions and improve the validity of our experimental design. Additionally, the IBS could diminish when transitioning from face‐to‐face to remote interactions (Schwartz et al., 2022). The current study does not assert that the enhanced IBS achieved through pseudo‐mutual gazing is comparable to that observed in face‐to‐face interaction. The influence of other copresence factors merits further in‐depth investigation.

Furthermore, it is important to consider the potential impact of volume conduction effects (Aydore et al., 2013; Bruña et al., 2018) on interpreting the obtained IBS. To mitigate this issue, one could use data with bipolar settings or source‐space data. Several newer phase synchrony measures, such as corrected imaginary PLV (ciPLV) (Bruña et al., 2018) and weighted phase lag index (wPLI) (Vinck et al., 2011), have been proposed as alternatives to PLV. The results obtained using ciPLV and wPLI are presented in the Supporting Information section, where we examine the influence of dyadic gender composition, task mode, and their interaction effect on the IBS of these two measures. Further exploring the differences between these phase synchrony measures could provide valuable insights.

One previous fNIRS study (Li et al., 2021) demonstrated that male–male dyads exhibited stronger IBS than either female–female or female–male dyads. Additionally, another fNIRS study (Cheng et al., 2015) indicated that mixed‐sex dyads showcased greater IBS than same‐sex dyads. However, our study's findings diverged from these previous conclusions, as we detected no significant differences in IBS across various gender compositions. Interestingly, despite observing differences in behavioral performance between gender compositions, these disparities did not translate into our IBS results. These outcomes emphasize the need for further exploration, ideally with a larger pool of dyadic participants and within the context of more complex social interaction settings.

## 5 CONCLUSIONS

This study investigated an interaction environment that integrated a hyperscanning EEG system, eye trackers, and LSL‐based multimodal data streaming platform to explore physiological signatures of social interaction through interbrain couplings and eye movements. The proposed pseudo‐eye contact approach could share mutual gaze, enabling eye movement to be shown on the interacting partner's screen and facilitating remote partners to perform cooperative and cooperative activities efficiently. Additionally, low‐frequency interbrain couplings involving frontal and temporal areas changes during joint attention tasks, supporting that the shared gaze is a promising nonverbal channel.

## CONFLICT OF INTEREST STATEMENT

The authors declare that there are no conflicts of interest that could be perceived as prejudicing the impartiality of the research reported.

### PEER REVIEW

The peer review history for this article is available at https://publons.com/publon/10.1002/brb3.3181.

## Supporting information

Supporting Information

## Data Availability

The data that support the findings of this study are openly available in Open Science Framework (OSF) at http://doi.org/10.17605/OSF.IO/PQ9DG.
