# Supplementary material for: Pseudo‐mutual gazing enhances interbrain synchrony during remote joint attention tasking
Source: Brain Behav. 2023 Jul 26;13(10):e3181. doi: 10.1002/brb3.3181 (PMC10570487; doi:10.1002/brb3.3181)
Supplement: Supplementary file 1 — Supporting Information [file BRB3-13-e3181-s001.docx]

Supplementary Materials for

*Pseudo–Mutual Gazing Enhances Interbrain Synchrony During Remote Joint Attention Tasking*

Chun-Hsiang Chuang and Hao-Che Hsu

This study originally recruited a total of 60 participants, consisting of 47 females and 13 males. However, we had to discard one dyad’s data (consisting of 1 female and 1 male) due to equipment malfunction. Consequently, the remaining dyads were made up of 18 female-female, 10 female-male, and one male-male dyads. The detailed composition is presented in the table below.

Supplemental TABLE 1. Gender composition among dyads

| Dyads | female | male |
| --- | --- | --- |
| female | 18 |  |
| male | 10 | 1 |

|  |
| --- |
| Supplemental FIGURE 1. Effects of task mode and gender composition on ciPLV-based IBS. The terms FF and FM denote female-female and female-male dyads, respectively. |

|  |
| --- |
| Supplemental FIGURE 2. Effects of task mode and gender composition on wPLI-based IBS. The terms FF and FM denote female-female and female-male dyads, respectively. |
